# Health-Related Quality of Life and Preferred Health-Seeking Institutions among Rural Elderly Individuals with and without Chronic Conditions: A Population-Based Study in Guangdong Province, China

**DOI:** 10.1155/2014/192376

**Published:** 2014-05-18

**Authors:** Zhiheng Zhou, Caixia Wang, Huajie Yang, Xiang Wang, Chanjiao Zheng, Jiaji Wang

**Affiliations:** ^1^School of Public Health, Guangzhou Medical University, 195 Dongfeng Xi Road, Guangzhou 510182, China; ^2^Department of Internal Medicine, Guangzhou First People's Hospital Affliated to Guangzhou Medical University, Guangzhou 510180, China; ^3^Liyuan Hospital of Tongji Medical College, Huazhong University of Science and Technology, Wuhan 430077, China

## Abstract

The aim of this study was to examine health-related quality of life (HRQL) as measured by SF-36 and to identify these factors and the preferred health-seeking institutions of 12,800 persons aged 60 and older with and without chronic conditions in rural areas of Guangdong Province by multistage stratified cluster sampling method. HRQL among rural elderly subjects with chronic conditions was lower than that of elderly subjects with no chronic conditions. Multiple linear regression showed that marital status, living with children, cardiovascular disease, osteoporosis, cataract disease, and mental disease were the main affecting factors of HRQL. The main preferred health-seeking institutions selected by the rural elderly were community/town health service institutions, district hospitals, or secondary hospitals. Our findings indicate that the elderly in rural areas of Guangdong Province have a poor HRQL and incorrect health-seeking pathway. The healthcare policymakers should emphasize the need of developing effective and targeted community services strategies to improve the elderly individuals' HRQL in rural areas of China.

## 1. Introduction 

The proportion of older adults is increasing in most developing countries, which has become an important concern internationally [1]. As a result of China's overall economic environment and the accelerated process of urbanization, the number of elderly individuals is increasing, especially in some rural districts [2]. Rural elderly people often live in worse socioeconomic conditions and have poorer literacy skills compared with their urban counterparts [3]. Therefore, their social and physical well-being has become a challenging issue in China. China will have to institute very rapid reforms to care for an increasingly older society, given its relatively limited social security system. Previous studies have developed patient-report measures for exploring HRQL [4–8]. Many Chinese researchers have explored indicators relating to elderly people [2, 9–11] including HRQL. Li et al. [12] examined the association between living arrangements and health among very elderly Chinese and found that having a spouse in the household provided the best health protection, while living alone and living with children were associated with both health advantages and disadvantages. Reports found that elderly subjects had lower scores on a range of indicators relating to physical and mental health and social interaction. However, little is known about whether these findings are the same for older people, living in rural areas of China.

Chronic diseases are the leading health challenges of the 21st century. The World Health Organization (WHO) reported that, out of 36 million deaths that occurred in 2008, almost two-thirds were due to chronic conditions, such as cardio-cerebral-vascular disease, hypertension, chronic respiratory disease, and diabetes. Nearly 80% of these deaths occurred in middle-income and low-income countries, populations, and communities [13, 14]. Chronic diseases have become a major public health problem in China, where nearly 260 million chronic disease cases have recently been diagnosed. Along with the accelerating process of industrialization, urbanization, and aging, China's health system will face multiple challenges from the increasing prevalence of chronic disease and mortality rates and from the increasing incidence of these conditions among younger and lower-income individuals [15, 16]. Chronic diseases not only pose a heavy economic burden on the individual, family, and society, but also seriously affect patients' quality of life. Studies performed during the past few decades have indicated that health-related quality of life (HRQL), which refers to physical, psychological, and social functioning as reported by the patients themselves, has become an important component of chronic disease evaluation and monitoring in addition to conventional objective indicators, such as morbidity, mortality, and clinical measurements [17]. The questionnaires, including the SF-36, EuroQol questionnaire (EQ-5D), SF-12, the Health Utilities Index (HUI), and other measurement systems, have been used in large surveys to measure HRQL in both general and specific populations [18, 19]. Many studies [20, 21] have examined the association of chronic conditions and HRQL as defined by the physical component summary (PCS) and the mental component summary (MCS) scores of the widely used SF-36 health survey [22–24], but few reports have studied that for the rural elderly in China.

The Chinese government has built a community healthcare services network and trained the GP as “gatekeeper” for community health since 1997. The first-treatment system pilot project was carried out in social CHS institutions in Beijing, Shenzhen, Nanjing, Nanning, and other cities since 2006 in China [10]. However, the initial effects of community healthcare services network were discouraging. Currently, there is no well-established unified first-treatment system in community and relevant supporting medical insurance system in Guangdong Province. With no mandatory guide on health-seeking pathway, people face multiple choices when selecting which medical institutions to visit. The long-standing dual framework of city and countryside leads to the unequal distribution health resources between urban and rural areas.

In this study, the rural elderly population of Guangdong was profiled to establish a baseline for future evaluation and surveillance. We examined health-related quality of life (HRQL) as measured by SF-36 to identify these factors and the preferred health-seeking institutions of persons aged 60 and older with and without chronic conditions in rural areas of Guangdong Province, China.

## 2. Methods

### 2.1. Location

This was a cross-sectional survey of a random sample of older adults selected from using a stratified, multistage sampling method. In the first stage, to ensure the sample representativeness in the terms of demographic and socioeconomic status indicators, Guangdong Province was divided into central Guangdong, east Guangdong, and west Guangdong. These three regions are not only representative of Guangdong with respect to geographical scale and distribution, but also representative of various types of industry in Guangdong. In the second stage, 17 out of 45 counties in Guangdong Province were randomly selected, which included 7 counties in central Guangdong, 5 counties in east Guangdong, and 5 counties in west Guangdong. In the third stage, 5 townships (known as xiang and zhen, respectively, in Chinese) were randomly selected from each of the second-stage samples. Therefore, central Guangdong was typical of well-developed regions and had 35 townships; east Guangdong was representative of moderately developed regions and had 25 townships; and west Guangdong was representative of underdeveloped regions and had 25 townships. These three regions are representative of the population with respect to lifestyle and geographical distribution. Therefore, a survey of older adults from these regions could well represent the HRQL status and the preferred health-seeking institutions of the rural elderly in Guangdong.

### 2.2. Subjects

In this study, the estimated sample size was 12,600 according to the survey sample rate (1/500), rural older population size of Guangdong Province (6.3 million). The entire Guangdong Province was broken down into three strata: east Guangdong, west Guangdong, and central Guangdong, and, in each stratum, the townships were selected for the survey by multistage stratified cluster sampling method. More than 150 rural older residents were involved in each survey townships and a total of 12,800 residents were investigated in our study. This survey was based on the local authority register for individuals born before 31th July 1952. Eligibility criteria were (i) residence in one of the three regions and (ii) aging 60 or older. Participants answered questions concerning sociodemographic, physical, and mental health and their preferred health-seeking institutions. If the individuals had limited autonomy, such as physical limitations or mental incapacity, their family members or close relatives could act as their proxy in accepting the invitation and helping to complete the questionnaires. In the target townships, during the time of the study, all individuals' records were obtained from the Sixth Nationwide Census, and the eligible individuals were invited to participate in the study.

### 2.3. Classification of Chronic Conditions

The diagnoses and morbidity data were coded according to ICD-10 and the disease classification of the fourth National Health Service Survey (NHSS) of China. Twenty-two chronic diseases were extracted from the codes on the basis of both prevalence and disease burden as follows: cardiovascular (hypertension, heart attack in the last year, angina, or coronary artery disease, other heart conditions, such as problems with heart valves or the rhythm of your heartbeat), endocrine (diabetes or high blood sugar), respiratory (chronic obstructive pulmonary disease (COPD)), musculoskeletal (rheumatoid arthritis (RA), osteoarthritis, degenerative arthritis, and osteoporosis), haematopoietic (anemia), gastrointestinal (stomach disease, such as gastritis or duodenitis, kidney disease, and liver disease, such as hepatitis B or C), ENT disease (nasal allergies, cataract disease), other physical health (stroke, migraine headaches, chronic fatigue syndrome or fibromyalgia, and cancer, except skin cancer), and mental health (depression, anxiety, alcohol, or other substance use disorders). Conditions were numbered based on the first reported chronic condition, which was considered the most important one as defined by the NHSS questionnaire.

Based on checklist responses, four “morbidity” groups were created for comparisons relevant to the primary study objectives.

1. Healthy: no reported chronic conditions are related to physical or mental health.
2. Physical health condition: 1 or more chronic physical conditions, but none, are related to mental health.
3. Mental health condition: 1 or more chronic mental health conditions, but none, are related to physical health.
4. Physical and mental health (comorbid) condition: one or more chronic physical conditions and one or more conditions are related to mental health.

### 2.4. Instruments

The following instruments were used:

1. demographic researcher-developed questionnaire measuring sociodemographic factors, including sex, age, marital status, yearly income, educational level, and chronic diseases;
2. short form-36 health survey questionnaire (SF-36). Although the SF-36 is American in origin, the Chinese version has good reliability and validity and is appropriate for the evaluation of HRQL in an elderly Chinese population [25]. The SF-36 consists of 36 items representing eight generic health concepts: physical functioning (PF) (10 items); social functioning (SF) (two items); role limitations due to physical problems (RP) (four items); role limitations due to emotional problems (RE) (three items); mental health (MH) (five items); vitality (VT) (four items); bodily pain (BP) (two items); and general perception of health (GH) (five items). Higher raw scores indicate better health. Subscale scores were combined and transformed into scores of 0 to 100 for physical and mental functioning and presented as the mental component summary scale (MCS) and physical component summary scale (PCS). The domains of PF, RP, BP, and GH correlate most highly with the PCS, while the MH, RE, VT, and SF correlate most highly with the MCS.
3. Based on a large sample of adults, the two extensively documented component summary measures are both scored on the T-score metric, with a mean of 50.0 (and a standard deviation of 10.0). As with the prior version of the SF-36, one important aspect of the SF-36 constructed validation procedures included known group comparisons. These analyses demonstrated the ability of the PCS and MCS measures to discriminate groups of individuals with only a physical or a mental health condition from a “healthy” group and from groups with comorbid (physical and mental health) conditions [26];
4. preferred health-seeking institutions were also ascertained including which institutions among community health service centers, district hospitals or secondary hospitals, and tertiary general hospitals or special hospitals will be selected as the first-treatment institutions when they need healthcare, the reasons for selecting or existing CHS as the first-treatment institutions.

### 2.5. Quality Control

Subjects were contacted first by letter and then by follow-up telephone call to explain the study. After giving informed consent, the subjects' ages were confirmed using the household registration system. Approval of the village council was sought, accompanied by cooperation of the town health center. During the face-to-face field survey, trained research assistants explained how to fill in the questionnaires and helped participants complete them in their home, with the procedure lasting 25 minutes on average. The survey was conducted between August 2012 and February 2013. Questionnaires that were less than 80% complete were rejected. The database was established by EpiData3.1 (EpiData Association, Odense, Denmark), and double input was conducted to ensure accuracy.

### 2.6. Statistical Analyses

After checking the questionnaire, Epidata 3.1 software was used for the data entry procedure. To ensure the accuracy of data entry, double-entry method was adopted. SPSS 13.0 statistical software (Version13.0; SPSS Inc., Chicago, IL, USA) was applied for statistical analysis. The data are presented as mean values ± standard deviation. Differences of the mean values between groups were analyzed with* t*-test or analysis of variance. Counting data are presented with rate or composition ratio and the differences between groups were compared with chi-square test. Multiple linear regression model was used to determine which chronic diseases affected the older adults' HRQL. Pearson's correlative analysis was conducted to assess the association between HRQL score and preferred health-seeking institutions. *P* ≤ 0.05 was considered statistically different.

### 2.7. Ethical Considerations

The Ethics Committee of Guangzhou Medical University approved the study, and the research proposal was submitted and approved by the Ethical Review Committee in the target region (The Ethics Committee of Guangdong Medical Doctor Association). Oral informed consent was obtained from each participant. Participants were assured of their right to refuse to participate or to withdraw from the study at any time. Anonymity and confidentiality of the participants were assured. Participants were presented with a small gift (valued 1.5 USD) on completion of the survey.

## 3. Results

### 3.1. Participant Characteristics

As shown in Table 1, the majority of the 12,800 study participants were males (47.7%) and females (52.3%) were represented almost equally. The age range of the participants was between 60 and 92 years, with a mean of 71.2 years. Approximately equal percentages reported their educational level as illiterate (56.4%), primary (28.9%), secondary (11.8%), and university (2.9%). In addition, 55.3% of participants were married/living with partner, 10.2% were separated or divorced, 6.4% were never have been married, and 58.8% were farmers. The numbers of the elderly in central, east, and west Guangdong were 5222 (40.8%), 3930 (30.7%), and 3648 (28.5%), respectively. A total of 2,893 participants were included in the healthy group, 6,234 in the physical health condition, 1,510 in the mental health condition, and 2,163 in the physical and mental health condition (comorbidity) groups.

### 3.2. Reporting Frequency of Specific Conditions

Overall, the average number of chronic conditions reported was 3.1 (SD = 2.6). The five most commonly reported ones were hypertension (*n* = 3776, 29.5%), cataract disease (*n* = 3635, 28.4%), stomach disease (*n* = 2138, 16.7%), kidney disease (*n* = 2125, 16.6%), and osteoarthritis, degenerative arthritis (*n* = 2074, 16.2%).  Table 2 provides a list of chronic conditions included in the present study, with corresponding frequency of self-report for both males and females. Considering all conditions included in the analysis, women reported having more conditions than did men (mean = 3.4 (SD = 2.9) for women and mean = 2.8 (SD = 2.5) for men) (*P* = 0.000). As expected, there were differences between males and females in the ordering of conditions by frequency with which they were reported (*P* = 0.000). Migraine was more common in women (20.4%) than in men (7.4%) (*P* = 0.000), as was anxiety (18.0% for women versus 11.4% for men, *P* = 0.000), depression (17.5% for women versus 10.2% for men, *P* = 0.000), osteoporosis (15.8% versus 7.2%, *P* = 0.000), chronic fatigue syndrome (7.2% for women versus 1.8% for men, *P* = 0.000), and anemia (5.3% for women versus 2.1% for men, *P* = 0.000). Hypertension was more common in men (32.9%) than in women (27.7%, *P* = 0.000), as was other heart conditions (15.9% for men versus 12.4% for women, *P* = 0.000), nasal allergies (13.4% for men versus 10.3% for women, *P* = 0.000), cataract disease (32.1% for men versus 25.6% for women, *P* = 0.000), liver disease (14.2% for men versus 10.4% for women, *P* = 0.000), and alcohol or other substance use disorders (5.2% for men versus 1.2% for women, *P* = 0.000). Approximately 35.7% (*n* = 3537) reported having a single condition (physical or mental health), 30.4% (*n* = 3012) reported having 2, 16.2% (*n* = 1605) reported having 3, and the remainder (17.7%, *n* = 1753) had 4 or more conditions. Among those reporting a single condition, approximately 17.5% (*n* = 619) reported having 1 single mental health condition. For those with 2 conditions, 69.0% (*n* = 2078) had 2 physical conditions and 6.0% (*n* = 181) had 2 mental health conditions. The remainder (25.0%) reported having 1 physical and 1 mental health condition.

### 3.3. HRQL of Rural Elderly Subjects in Guangdong Province

Table 3 showed that the HRQL scores varied from 41.2 ± 19.3 to 82.4 ± 17.2. The RP and RE scores were the lowest among the eight dimensions of the SF-36, with PF score being the highest. Among the eight generic health of SF-36, all of the HRQL scores in the no chronic condition elderly group were higher than those of chronic condition (*P* < 0.05). For PF, the subjects expressed difficulty in understanding the concept of walking distance. Although “mile” had been replaced in the Chinese version of the SF-36 by “kilometer,” the rural elderly tended to interpret it as the Chinese measure “Li,” which means “0.5 kilometer.” The elderly in this study scored relatively higher on PF compared with the original US validation sample, which is consistent with reports by rural elderly adults that walking is considered one of the easiest activities in their daily lives. Regarding items measuring “accomplishment” in RP and RE, subjects explained that the demands of their job as a farmer varied. During quiet periods, they often were unoccupied, whereas, during busy seasons, they felt that they had to accomplish whatever was necessary regardless of their level of motivation.

### 3.4. HRQL Burden of Chronic Physical Health Conditions

Relative to participants in the healthy group, those in the physical health condition group showed substantial decrements in PCS scores. On average, males in the physical health condition group scored 6.6 points lower (95% confidence interval (CI) [−7.5, −5.6]) on PCS than did their healthy counterparts. Similarly, females in the physical health condition group scored 7.7 points lower (95% CI [−8.8, −6.6]) than did females defined as healthy. Relative to their PCS scores, decrements in MCS for the physical health condition group were smaller for both males and females. Compared to scores for healthy participants, MCS scores for males and females in the physical health condition group were 1.9 points (95% CI [−2.9, −0.9]) and 2.3 points (95% CI [−3.5, −1.1]) lower, respectively. These results are shown in Table 4.

### 3.5. HRQL Burden of Chronic Mental Health Conditions

The MCS scores for participants in the mental health condition group revealed large decrements compared to scores for the healthy group. Decrements were similar for males (11.5 points lower; 95% CI [−13.5, −9.5]) and females (12.1 points lower; 95% CI [−14.9, −9.2]). For both males and females, PCS scores of those in the mental health condition were not meaningfully different than PCS scores of those in the healthy group. Scores were 0.9 points lower (95% CI [−2.8, 1.4]) for males and 0.5 points higher (95% CI [−2.7, 3.3]) for females (Table 4).

### 3.6. Physical Health Conditions: Incremental HRQL Burden of Mental Health Comorbidity

Table 4 shows that, for those in the physical health condition group, a comorbid mental health condition decreased PCS scores by an additional 4.7 points (95% CI [−6.1, −3.4]) (males) and 3.9 points (95% CI [−5.1, −2.7]) (females). Thus, total reductions were 11.1 and 11.4 points compared to the healthy group. The incremental MCS burden of a mental health comorbidity to physical health conditions alone was more pronounced, with increased decrements of −13.8 for males (95% CI = [−15.2, −12.4]) and −13.3 for females (95% [−14.5, −12.1]). For both males and females, total MCS burden was approximately 15.2 points when the comorbid group was compared to the healthy group.

### 3.7. Mental Health Conditions: Incremental HRQL Burden of Physical Health Comorbidity

Finally, Table 4 shows that, relative to participants in the mental health condition group, those reporting both a mental hHealth and a physical health condition experienced lower HRQL as assessed by both PCS and MCS scores. PCS scores were 10.4 points lower (95% [−12.5, −8.3]) for males and 11.5 points lower (95% CI [−14.5, −8.8]) for females. Compared to those categorized as having a mental health condition only, average additional decrements in MCS were 4.2 points (95% CI [−6.4, −2.0]) and 3.5 points (95% CI [−6.4, −0.6]) (resp.) for males and females.

### 3.8. Factors Influencing HRQL among Elderly Subjects

Multiple linear regression was conducted to determine the factors affecting HRQL. Nine variables were included in the regression model. Cataract disease (beta = −0.589) was the most important factor related to HRQL. This was followed by cardiovascular disease (beta = −0.589), marital status (beta = −0.418), osteoporosis (beta = −0.327), mental disease (beta = −0.249), living with children (beta = 0.231), economic status (beta = 0.149), educational level (beta = 0.147), and age (beta = −0.146) (Table 5). Chronic diseases had a major impact on the HRQL of rural elderly subjects. Cataract disease and cardiovascular disease remain as the important diseases affecting the quality of life of the elderly in rural areas of China.

### 3.9. The Preferred Health-Seeking Institutions of the Rural Elderly

Table 6 shows that the preferred health-seeking institutions selected by the rural elderly when they had medical needs included community/town health service (CHS) institutions (40.8%), district hospitals or secondary hospitals (DH/SH) (49.4%), and tertiary general hospitals or special hospitals TGH/SH (9.8%). The healthy rural elderly selected the (CHS) institutions (43.8%) as the preferred health-seeking institutions more than that of the rural elderly with chronic conditions (37.1%) when they felt unwell initially, felt abnormal, or were suffering from common diseases (*P* = 0.000), while the rural elderly with chronic conditions selected the DH/SH (55.8%) and TGH/SH (7.1%) as the preferred health-seeking institutions more than that of the healthy rural elderly (50.7% and 5.5%) (*P* = 0.000). HRQL and preferred health-seeking institutions among this elderly population were significantly correlated (*r*_CHS institutions_ = 0.204, *P* = 0.003; *r*_DH/SH_ = −0.314, *P* = 0.002; *r*_TGH/SH_ = −0.431, *P* = 0.000).

### 3.10. The Reasons for Selecting or Not Selecting CHS Institutions by the Rural Elderly

Table 7 shows that the main reasons why the rural elderly selected the community/town health service institutions as the preferred health-seeking institutions were short distance (72.17%), good attitude for service (35.42%), convenient procedures (26.71%), reliable medical skills (25.43%), short time for waiting (17.82%), low price (16.72%), and so forth. When we investigated the reasons why rural elderly did not visit the CHS institutions for treatment, compared to the DH/SH and the TGH/SH, we found that they focused on four factors: lack of drug variety (28.30%), obsolete equipment (31.42%), doubt about the medical skills of the staff (16.53%), and an uncomfortable consulting environment (9.45%). These issues also were accountable for the issues that existed in CHS institutions compared to the DH/SH and the TGH/SH.

## 4. Discussion

The goal of this study was to examine the burden of self-reported chronic physical and/or mental health conditions on each of the two commonly used SF-36 summary measures of HRQL. Our results showed that frailty in older subjects had marked negative effects on the eight dimensions of the SF-36. Managing elderly people with chronic disease is complex because they commonly have multiple chronic conditions. Effective chronic disease management requires recognition of this complexity because there may be conflicts among management guidelines for the multiple conditions present [27]. Older adults in rural areas of Guangdong Province often have difficult pressures in life, including chronic diseases, mental and physical disorders, serious psychological fatigue, and psychological problems. The mental and physical disorders may in turn increase stress and lead to poor health and chronic diseases [28]. In the present study, hypertension, cataract disease, stomach disease, kidney disease, and osteoarthritis, degenerative arthritis, were the major conditions for the rural elderly subjects in Guangdong Province, and the cardiovascular disease, osteoporosis, cataract disease, and mental disease were the major conditions that impacted HRQL.

This study showed the mental component summary (MCS) score and the physical component summary (PCS) score. Consistent with findings of prior studies, the impact of at least 1 chronic physical condition for this sample of adults in the general population was reflected to a greater extent in PCS rather than in MCS scores [29, 30]. Similarly, the impact of at least 1 chronic mental health condition was primarily reflected in MCS rather than in PCS.

Relative to individuals with only physical health conditions, males and females reporting ≥1 comorbid mental health condition showed (on average) an additional PCS decrement of 4.7 and 3.9 points, respectively. These values are larger than 2 points, which is the recommended minimally important difference (MID) for group comparisons [26]. The finding that a comorbid chronic mental health condition was associated with an additional HRQL decrement is consistent with those reported in prior studies [22, 23]. One of the strengths of the present study is that it provides quantitative information on specific PCS and MCS decrements associated with a comorbid condition related to mental health, as well as a comorbid condition related to physical health.

Compared to having a mental health condition only, the presence of ≥1 comorbid physical health condition led to further MCS decrements of 3.3 points for females and 4.0 points for males. Both values are above the recommended MID for MCS, which is 3 points [26]. The observed incremental impact of a physical comorbidity on MCS and the incremental impact of mental health comorbidity on PCS scores were robust across groups organized by “physical disease clusters.” Total decrements in PCS scores associated with a chronic physical condition and a comorbid mental health condition were greater for the musculoskeletal, endocrine, and respiratory disease clusters (15.2 to 16.8 points) than for the gastrointestinal or cardiovascular disease clusters (11.9 to 12.2 points). This study indicated that we should focus on the elderly in rural areas, to formulate effective measures to improve QoL. Chronic diseases were also associated with lower QoL and perceived QoL was significantly correlated with self-rated health [24, 31].

In accordance with earlier studies [32, 33], education was a significant positive contributor to overall HRQL. According to Lasheras et al. [34], lower educational level is associated with unhappiness, poor social relationships, poor self-assessed health, and sensory problems among the elderly. Education is an important indicator that may directly or indirectly influence HRQL through its association with higher social class and economic status. Our survey suggests that educational level is associated with better HRQL among rural elderly subjects. This is consistent with other studies conducted in Nigeria and Iran [33, 35], where older adults with better education generally enjoy higher incomes and better social support. Medical costs are a huge burden for elderly subjects because there is no universal health insurance in China and may have a greater impact than in developed countries. Both education and income have a major influence on the QoL of the elderly in China. In our study, subjects reported higher physical functioning compared with perceived physical and emotional limitations, as reflected by scores on the SF-36. Furthermore, elderly people being able to live with their families are very important to health outcomes and have a positive effect on QoL [36]. Good social relationships (including relationships with relatives and friends) are the most commonly reported factor influencing QoL in the elderly [37, 38].

In China, the government wants to build a community healthcare services network for people to receive CHS within 15 minutes. The government hopes that the CHS institutions will be the preferred health-seeking institutions selected by the rural elderly when they had medical needs and GP as “gatekeeper” for health. However, this study showed that the percentage of choosing third-class comprehensive hospital, specialized hospital, area hospital, or second-class hospital was large among the rural elderly, while choosing a CHS institution was less than 50%. The main reasons why the rural elderly did not select CHS institutions for treatment were focused on four aspects: lack of drug varieties, obsolete equipment, doubt in the medical skills of the staff, and an uncomfortable consulting environment. This showed that the Chinese government should invest more resources including the human resources, especially in rural and remote areas, and increase the drug choices, equipment to the CHS institutions, in order to improve their accessibility. It is necessarily and effectivily [39, 40] proven by the international studies.

Our use of data from three regions in Guangdong Province limited the applicability of our results to other provinces in China. Despite this, the results of the analysis provide an overall picture of the HRQL and preferred health-seeking institutions among older adults in rural Guangdong Province, which may facilitate further prospective studies.

## 5. Conclusions

Our findings indicate that the elderly in rural areas of Guangdong Province have a poor HRQL and incorrect health-seeking pathway. The specific PCS and MCS score decrements associated with a comorbid condition related to mental health, as well as a comorbid condition related to physical health. These results may have significant implications for healthcare policymakers, since they emphasize the need of developing effective and targeted community services strategies to improve the elderly individuals' HRQL in rural areas of China. Health authorities in China and community health centers need to provide these older people with adequate interventions, such as health education, health promotion, and health resources. It is recommended that all relevant stakeholders prioritize health promotion programs for the elderly and allocation of resources. In doing so, HRQL among the rural elderly in China can be improved.

## Figures and Tables

**Table 1:** Sample characteristics by “morbidity group.”

	Healthy (*n* = 2,893)	Physical (*n* = 6,234)	Mental (*n* = 1,510)	Physical and mental (*n* = 2,163)	Total sample (*n* = 12,800)
Sex					
Female	21.8	46.4	14.8	17.0	52.3
Male	24.6	51.7	8.4	15.3	47.7
Age					
60–64	32.7	40.2	8.6	18.5	30.1
65–69	25.5	46.9	11.2	16.4	27.3
70–74	18.1	55.8	13.4	12.7	21.8
≥75	10.1	61.7	13.3	14.9	20.8
Site in Guangdong Province					
Central	25.1	45.7	14.2	15.0	40.8
East	22.4	46.9	12.8	17.9	30.7
West	20.9	52.8	10.4	15.9	28.5
Education					
Illiterate	19.5	43.5	13.5	22.5	56.4
Primary	19.1	49.5	14.5	15.9	28.9
Secondary	24.7	51.2	10.3	13.8	11.8
University	26.2	48.1	11.4	14.3	2.9
Marital status					
Married/living with partner	27.1	45.8	10.6	16.5	55.3
Widowed	13.4	57.1	15.4	14.1	28.1
Separated/divorced	14.1	49.6	13.1	23.2	10.2
Never married	16.7	54.7	11.2	17.4	6.4
Economic status					
Poor	9.4	60.5	8.6	21.5	24.8
Intermediate	25.8	46.9	12.8	14.5	45.4
Good	31.4	41.2	11.7	15.7	29.8
Occupation					
Farmer	26.4	44.7	11.1	18.7	58.8
Other work	20.1	48.6	9.4	21.9	11.2
No work	17.9	53.9	14.5	13.7	30.0

Total	22.6	48.7	11.8	16.9	100.0

**Table 2:** Percent of participants reporting each condition from the chronic condition checklist.

Chronic condition	Total sample	Males	Females	*P* value
Cardiovascular				
Hypertension	29.5	32.9	27.7	0.000
Heart attack in the last year	1.6	1.9	1.2	0.061
Angina or coronary artery disease	7.4	8.1	6.9	0.167
Other heart conditions, such as problems with heart valves or the rhythm of your heartbeat	14.6	15.9	12.4	0.000
Endocrine				
Diabetes or high blood sugar	9.4	10.5	8.1	0.072
Respiratory				
Chronic obstructive pulmonary disease (COPD)	7.9	8.6	6.8	0.123
Musculoskeletal				
Rheumatoid arthritis (RA)	9.2	7.6	10.8	0.054
Osteoarthritis, degenerative arthritis	16.2	17.5	15.1	0.081
Osteoporosis	12.2	7.2	15.8	0.000
Haematopoietic				
Anemia	3.8	2.1	5.3	0.000
Gastrointestinal				
Stomach disease, such as gastritis or duodenitis	16.7	16.5	16.9	0.541
Kidney disease	16.6	17.7	15.4	0.003
Liver disease, such as hepatitis B or C	12.1	14.2	10.4	0.000
ENT disease				
Nasal allergies	12.5	13.4	10.3	0.000
Cataract disease	28.4	32.1	25.6	0.000
Other physical health				
Stroke	5.6	6.4	4.1	0.064
Migraine headaches	13.4	7.4	20.4	0.000
Chronic fatigue syndrome or fibromyalgia	4.8	1.8	7.2	0.000
Cancer, except skin cancer	1.2	1.3	1.1	0.327
Mental Health				
Depression	13.4	10.2	17.5	0.000
Anxiety	14.7	11.4	18.0	0.000
Alcohol or other substance use disorders	3.4	5.2	1.2	0.000

**Table 3:** HRQL of the rural elderly subjects in Guangdong Province.

Short Form-36	Total sample	Chronic condition	No chronic condition	*P* value
PF (physical functioning)	82.4 ± 17.2	76.2 ± 16.4	83.3 ± 21.6	0.002
RP (role functioning physical)	41.2 ± 19.3	37.6 ± 8.5	48.7 ± 16.9	0.000
BP (bodily pain)	59.6 ± 16.8	51.1 ± 12.6	63.4 ± 17.8	0.000
GH (general health perceptions)	57.3 ± 14.5	53.6 ± 12.5	60.4 ± 14.8	0.001
VT (vitality)	55.2 ± 18.6	52.4 ± 10.6	60.1 ± 12.6	0.000
SF (social functioning)	59.6 ± 19.4	56.4 ± 9.5	61.9 ± 13.1	0.007
RE (emotional role functioning)	43.5 ± 12.5	41.6 ± 7.3	51.6 ± 9.7	0.000
MH (mental health)	60.2 ± 20.7	57.5 ± 10.7	62.9 ± 12.2	0.006

**Table 4:** Mean PCS and MCS decrements associated with a chronic physical health condition, a chronic mental health condition, or comorbid conditions (all are compared to healthy reference group).

	Incremental decrement	Males	Incremental decrement	Females
		Total decrement		Total decrement
PCS				
≥1 chronic physical health condition only	−6.6 (−7.3, −5.6)	−11.1 (−12.5, −9.8)	−7.7 (−8.6, −6.6)	−12.4 (−12.7, −10.1)
Addition of ≥1 chronic mental health condition	−4.7 (−6.1, −3.4)		−3.9 (−5.1, −2.7)	
≥1 chronic mental health condition only	−0.9 (−2.8, −1.4)	−11.1 (−12.5, −9.8)	−0.5 (−2.7, −3.3)	−12.4 (−12.7, −10.1)
Addition of ≥1 chronic physical health condition	−10.4 (−12.5, −8.3)		−11.7 (−14.5, −8.8)	
MCS				
≥1 chronic physical health condition only	−1.9 (−2.9, −0.9)	−15.5 (−16.9, −14.0)	−2.3 (−3.4, −1.2)	−15.4 (−16.8, −14.3)
Addition of ≥1 chronic mental health condition	−13.8 (−15.2, −12.4)		−13.3 (−14.5, −12.1)	
≥1 chronic mental health condition only	−11.5 (−13.5, −9.5)	−15.5 (−16.9, −14.0)	−12.1 (−14.9, −9.2)	−15.4 (−16.8, −14.3)
Addition of ≥1 chronic physical health condition	−4.2 (−6.4, −2.0)		−3.5 (−6.4, −0.6)	

**Table 5:** Factors influencing HRQL among the rural elderly subjects in Guangdong Province.

Variables	B	SE	Beta	*t*	*P* value
Age	−0.155	0.158	−0.146	−4.269	0.002

Educational level	1.915	0.237	0.147	8.083	0.000

Economic status	3.236	0.347	0.149	4.912	0.000
Marital status	−0.429	0.267	−0.418	−4.167	0.004
Living with children.	3.157	0.386	0.231	5.096	0.000
Cardiovascular disease	−0.312	0.289	−0.429	−0.423	0.003
Osteoporosis	−0.326	0.339	−0.327	−5.063	0.000
Cataract disease	−0.526	0.409	−0.589	−2.653	0.024
Mental disease	−0.516	0.246	−0.249	−3.268	0.017

**Table 6:** Preferred health-seeking institutions of the rural elderly subjects in Guangdong Province.

	Total sample	Chronic condition	No chronic condition	*P* value
Community health service	40.8	37.1	43.8	0.000
District hospitals or secondary hospitals	49.4	47.8	50.7	0.000
Tertiary general hospitals or special hospitals	9.8	15.1	5.5	0.000

**Table 7:** Reasons for selecting or not selecting CHS as the first treatment institutions among the rural elderly subjects in Guangdong Province.

The reasons for selecting CHS institutions	%	The reasons for not selecting the CHS institutions	%
Appointed healthcare facilities	14.51	Lack of drug varieties	28.30
Good attitude for service	35.42	Obsolete equipment	31.42
Short distance	72.17	Doubtful for medical skills	16.53
Reliable medical skills	25.43	Uncomfortable consulting environment	9.45
Convenient procedures	26.71	Inconvenient procedures	4.41
Short time for waiting	17.82	Bad attitude for service	3.75
Low price	16.72	High price	2.63
Comfortable consulting environment	6.91	Nonappointed healthcare facilities	2.41
Be familiar with doctors and nurses	15.83	Else	2.37
Else	6.5		
